# Xenobiotic metabolism and its physiological consequences in high-Antarctic Notothenioid fishes

**DOI:** 10.1007/s00300-021-02992-4

**Published:** 2021-12-26

**Authors:** Anneli Strobel, Roger Lille-Langøy, Helmut Segner, Patricia Burkhardt-Holm, Anders Goksøyr, Odd André Karlsen

**Affiliations:** 1 Alfred Wegener Institute Helmholtz Centre for Polar and Marine Research, Am Handelshafen 12, 27570 Bremerhaven, Germany; 2 Department of Biological Sciences, University of Bergen, Thormøhlensgate 53B, 5006 Bergen, Norway; 3 Department of Infectious Diseases and Pathobiology, Centre for Fish and Wildlife Health, University of Bern, Länggassstrasse 122, 3012 Berne, Switzerland; 4 Man-Society-Environment, University of Basel, Vesalgasse 1, 4051 Basel, Switzerland; 5 Institute of Marine Research, P.O. Box 1870, Nordnes, 5817 Bergen, Norway

**Keywords:** Notothenioids, Aryl hydrocarbon receptor, Polycyclic aromatic hydrocarbons, Luciferase reporter gene assay, Hepatocyte metabolism

## Abstract

The Antarctic ecosystem is progressively exposed to anthropogenic contaminants, such as polycyclic aromatic hydrocarbons (PAHs). So far, it is largely unknown if PAHs leave a mark in the physiology of high-Antarctic fish. We approached this issue via two avenues: first, we examined the functional response of the aryl hydrocarbon receptor (Ahr), which is a molecular initiating event of many toxic effects of PAHs in biota. *Chionodraco hamatus* and *Trematomus loennbergii* served as representatives for high-Antarctic Notothenioids, and Atlantic cod, *Gadus morhua* as non-polar reference species. We sequenced and cloned the Ahr ligand binding domain (LBD) of the Notothenioids and deployed a GAL4-based luciferase reporter gene assay expressing the Ahr LBD. Benzo[*a*]pyrene (BaP), beta-naphthoflavone and chrysene were used as ligands for the reporter gene assay. Second, we investigated the energetic costs of Ahr activation in isolated liver cells of the Notothenioids during acute, non-cytotoxic BaP exposure. In the reporter assay, the Ahr LBD of Atlantic cod and the Antarctic Notothenioids were activated by the ligands tested herein. In the in vitro assays with isolated liver cells of high-Antarctic Notothenioids, BaP exposure had no effect on overall respiration, but caused shifts in the respiration dedicated to protein synthesis. Thus, our study demonstrated that high-Antarctic fish possess a functional Ahr that can be ligand-activated in a concentration-dependent manner by environmental contaminants. This is associated with altered cost for cellular protein synthesis. Future studies have to show if the toxicant-induced activation of the Ahr pathway may lead to altered organism performance of Antarctic fish.

**Supplementary Information:**

The online version contains supplementary material available at 10.1007/s00300-021-02992-4.

## Introduction

The Southern Ocean is progressively exposed to anthropogenic environmental influences, amongst them contamination by lipophilic organic contaminants including the persistent organic pollutants (POPs) such as dioxin-like compounds and polycylic aromatic hydrocarbons (PAHs). These contaminants are introduced to Antarctica via atmospheric long-range transport and global distillation processes, but also by local sources such as ships or research stations (Wania and Mackay 1996; IPCC 2014). Importantly, studies showed that the lipophilic contaminants can enter the Antarctic trophic food web, in which they bioaccumulate and biomagnify (Corsolini et al. 2002, 2003). Some studies already demonstrate that Antarctic fishes accumulate anthropogenic contaminants such as PAHs or halogenated aromatic hydrocarbons (HAHs), in both low- and high-Antarctic regions (Goutte et al. 2013; Strobel et al. 2016, 2018). The consequences for the Antarctic fauna, however, are still largely unknown (Bennett et al. 2015). The majority of the Antarctic fish species belong to the suborder of the Notothenioidei, which are endemic to the Southern Ocean (Eastman 2005; Matschiner et al. 2011). The stable, permanently cold and oxygen-rich waters, as well as low competition in this extreme environment, promoted unique evolutionary adaptations of the Notothenioids (Garofalo et al. 2009). One of the most prominent functional losses, for example, are the genetic mutations that caused losses of haemoglobin and myoglobin expression in the so-called ‘white-blooded icefishes’ (Channichthyidae) (Sidell and O'Brien 2006). Together with other physiological adaptations, such as the expression of antifreeze-glycoproteins and low metabolic rates, the icefish can only survive at constant and stable environmental conditions. Icefish are thus considered to be the most sensitive amongst the Antarctic fishes to the slightest of environmental perturbation (Barnes and Peck 2008; Beers and Sidell 2011). Another example for functional losses is the translocation of the mitochondrial genes encoding NADH dehydrogenase subunit 6 (ND6) in some high-Antarctic Notothenioids, which has considerable consequences for the amino acid sequence of the ND6 (Papetti et al. 2007; Mark et al. 2012). Even though physiological studies proved this translocation as functionally neutral, this example reflects the evolutionary peculiarities of Antarctic Notothenioids and the need to link findings at the gene expression level to the actual functionality of a protein.

Overall, Antarctic fish have successfully evolved numerous adaptations to their extreme as well as stable environment, but this may impose costs with respect to their capability to respond to changes in the environment. A molecular initiating event in the toxicity of many environmental contaminants, including dioxin-like compounds and PAHs, is ligand binding to and activation of the aryl hydrocarbon receptor (Ahr) (Denison and Nagy 2003).

The Ahr is a member of the family of basic helix-loop-helix (bHLH) Per-Arnt-Sim (PAS) transcription factors. The cytosolic form of this transcription factor is activated by the binding of ligands to the ligand binding domain (LBD) of the Ahr. After binding, it regulates the transcription of a number of biotransformation enzyme genes, such as cytochrome P450 1A (*cyp1a*) (King-Heiden et al. 2012). The expression or up-regulation of *ahr* and *cyp1a* are thus frequently used as biomarkers to assess the exposure of marine and freshwater fish to Ahr-binding contaminants (e.g. Sleiderink et al. 1995; Yamauchi et al. 2006; Jönsson et al. 2010; King-Heiden et al. 2012; Strobel et al. 2018). When it comes to Antarctic fish, only a handful of studies investigated the inducibility of *cyp1a* by xenobiotics in PAH exposed Notothenioids such as *Trematomus bernacchii* and *Chionodraco hamatus* (Focardi et al. 1995; Miller et al. 1998; Regoli et al. 2005). Just a single study focussed on the quantitative expression of *ahr* in contaminant-exposed Antarctic eelpout *Pachycara brachycephalum,* a non-Notothenioid fish species belonging to the deep-sea zoarcids (Strobel et al. 2018). However, the Ahr of the Notothenioids, which are highly endemic to the Southern Ocean, has not been characterized so far, especially not for its actual functionality.

Bearing in mind the evolutionary, functional losses and transactivation of genes found in Antarctic fishes, there is a need for demonstrating *ahr* gene expression to actually assess the capacity of xenobiotics to functionally activate the Ahr signalling pathway. As benchmark for the evolution of functional activation of Ahr signalling in Notothenioids, a comparison should be performed to Ahr activation in fish species from non-Antarctic regions. To assess the question of Ahr functionality in Antarctic Notothenioids, we used the well-established tool of reporter gene assays in the present study. These assays can reflect the binding to and activation of fish Ahr by xenobiotics in vitro*.* Importantly, it provides the possibility to qualitatively compare the capacities of the Ahr system between species (Billiard et al. 2002; Evans et al. 2005; Doering et al. 2015).

Beyond the evaluation of the molecular functionality of the Ahr pathway in Antarctic Notothenioids, the question remains which physiological and toxicological consequences could arise from this. The liver is a central organ in the Ahr-mediated metabolism of xenobiotics (Hinton et al. 2008), and thus, activation of the Ahr pathway is likely to be accompanied by metabolic costs. Some studies already report on an additional fraction of energy that is needed for detoxification processes, and is related to xenobiotics bioaccumulation (Ng and Gray 2011; Manciocco et al. 2014). Particulary for Antarctic fish, with their delicate energy resources and physiological specialization to the permanent cold (Pörtner 2006), such costs may be critical.

The present study aimed to test if organic pollutants leave a mark in the physiology of Antarctic fish, focussing at the molecular and cellular level. We hypothesize that Antarctic Notothenioids show functional losses of the cellular defensome—here, the Ahr, as it is the case with other elements of the defensome in Antarctic fish, e.g. the heat shock response, or the xenosensing pregnane X receptor (Pxr) in several fish species (Eide et al. 2018, 2021). To test this hypothesis, we analysed the presence of *ahr* transcripts of two high-Antarctic Notothenioids, sequenced the LBD and assessed by means of a GAL4-based luciferase reporter gene assay if the LBD of Ahr can be transactivated by BaP, a model PAH. We compared the BaP response with two additional compounds, chrysene and beta-naphthoflavone (BNF), and used Atlantic cod, *Gadus morhua,* as reference species. In order to gain insight into the metabolic costs of potential Ahr activation, we then performed respirometric measurements in isolated liver cells of the high-Antarctic fish, which were incubated under control conditions or under exposure to non-cytotoxic concentrations of BaP.

The two Antarctic species selected for this study were the red-blooded *T. loennbergii* and the white-blooded *C. hamatus*. Both species are benthic and they are widely distributed around the Antarctic continent between ~ 65 and ~ 76°S. Due to this distribution, they are classified as ‘high-Antarctic’ species (Gon and Heemstra 1990; Eastman 2005).

## Materials and methods

### Fish samples

Individuals of the high-Antarctic Notothenioids, *C. hamatus* and *T. loennbergii*, were caught by means of bottom trawls between 6th December 2015 and 14th February 2016 during cruise PS96 of the German research vessel *RV Polarstern* at 75° S, 30–38° W. *T. loennbergii* was caught at 750 m depths, *C. hamatus* at 300 m depths. Only fish which were in a good condition were taken from the trawls or the aquarium facilities on board and anaesthetized in 0.5 g*L^−1^ l tricaine methano-sulphonate (MS-222) before sampling.

Fish liver was either sampled for consecutive cell extraction, or snap-frozen in liquid nitrogen and stored at − 80 °C for further analysis.

### Test compounds as Aryl hydrocarbon receptor 2 agonists

The following compounds were used as Ahr2 agonists in the present study: BaP, beta-naphthoflavone (BNF) and chrysene. All compounds were dissolved in dimethyl sulfoxide (DMSO 0.01%) supplied by Sigma Aldrich.

### RNA preparation, cDNA synthesis, PCR and cloning of high-Antarctic fish Aryl hydrocarbon receptor

Total RNA was extracted from frozen liver tissue using a phenol–chloroform extraction. About 25–40 mg of frozen liver tissue was homogenized in 1 ml TriReagent® (Sigma-Aldrich, Buchs, Switzerland) in a TissueLyser (FastPrep-24™, MP Biomedicals, Lucerna Chem AG, Switzerland) twice with a duration of 45 s. The homogenization steps were performed under constant cooling with dry ice. After the phenol–chloroform extraction, the RNA was washed two times with ice-cold 75% ethanol. Afterwards, DNA was removed from the RNA samples using the DNA-*free*™ DNA Removal Kit (Ambion, life technologies, Thermo Fisher Scientific Inc.). RNA quality and integrity was verified by capillary electrophoresis (Bioanalyzer, Agilent Technologies, CA, USA). Only RNA samples of the best quality (RIN > 9.8) were used for the subsequent experiments.

For synthesis of double-stranded complementary DNA (cDNA), 0.5 µg of DNA-free RNA was reverse transcribed using the High Capacity cDNA Reverse Transcription Kit by Applied Biosystems (USA). The cDNA samples were purified with the DNA Clean & Concentrator™-5 kit, and the samples were stored at − 20 °C until analysed.

*Ahr* gene sequences are not available for *C. hamatus* or *T. loennbergii* in GenBank. Therefore, we designed primers located in conserved regions of piscine Ahr (bHLH and PAS-B), based on published sequence information in Karchner et al. (2005) and additionally on a putative Ahr sequence of the Antarctic fish *Notothenia coriiceps* (GeneBank accession no. #NW_011336470.1 and #XM_010778753; accession numbers are abbreviated with # from now on) (Table S1, supplementary). Using cDNA as a template, we amplified PCR fragments of the expected size (~ 900 bp), which were cloned (TOPO TA, Invitrogen Corporation, Carlsbad, California, USA) and sequenced (Microsynth AG, Balgach, Switzerland) afterwards.

### 5′-3′ rapid amplification of cDNA ends (RACE) PCR

5′-3′ RACE (FirstChoice® RLM-RACE Kit, Ambion Inc., USA) was performed according to the manufacturer’s protocol to obtain the full sequence of the Ahr. The gene-specific primers (GSPs) used for 5’-3’ RACE are given in Table S1. The PCR products were analysed on a 0.8% agarose gel, and positive products of ~ 800–900 bp length were extracted using the Zymoclean™ Gel DNA Recovery Kit (Zymo Research, California, USA). The purified PCR products were then cloned into pCR4-Topo® vectors (Invitrogen, Thermo Fisher Scientific Inc.). At least six plasmids per PCR product were purified with Plasmid Miniprep Kit by Zymo Research (Zymo Research Corporation, USA) and subsequently sequenced by Microsynth AG (Switzerland). The obtained sequences were verified by BlastN (ncbi.nlm.nih.gov) and analysed and assembled with CLC Main Workbench software (Qiagen, Denmark). The assembled sequences of the 5’-3’ RACE yielded a 1960 bp fragment for *C. hamatus* and a 3300 bp fragment for *T. loennbergii,* covering the bHLH, PAS-A and LBD including PAS-B. Further attempts to obtain the full-length Ahr sequences were not successful. Alignments were performed using CLC Main Workbench software (Qiagen, Denmark). The aligned amino acid sequences from seabream (*Sparus aurata)* Ahr1 #ABY82367, seabream Ahr2 #AAN05089, killifish (*Fundulus heteroclitus*) Ahr1 #O57452, killifish Ahr2 #AAC59696, goldfish (*Carassius auratus*) Ahr1 #ACT79400, goldfish Ahr2 #ACT79401, zebrafish (*Danio rerio*) Ahr1 #NP571103, zebrafish Ahr2 #NP571339, dogfish (*Mustelus canis*) Ahr1 #AAC60335, dogfish Ahr2 AAC60336, red seabream (*Pagrus major*) Ahr1 #BAE02821, red seabream *#*BAE02825, *C. hamatus* Ahr and *T. loennbergii* Ahr were used to construct a maximum likelihood phylogenetic tree using the neighbour-joining method (in CLC Main Workbench, Qiagen, Aarhus, Denmark).

The subsequent luciferase reporter gene assay was conducted with clones of the LBD from *C. hamatus* and *T. loennbergii*, corresponding to AA238-414 in zebrafish Ahr2; UniProt: #Q9YGV3). A genome analysis of the Notothenioids investigated herein was not covered within the framework of this study.

### Luciferase reporter gene assay

In vitro Ahr reporter gene assays, such as the luciferase reporter gene assay, are common methods for the semi-quantitative activation of PAHs and dioxin-like compounds in fish and have been used in several species from various environmental temperature regimes, and a feasible choice in case the full-length Ahr receptor is not available (Roy et al. 2011; Chao et al. 2012; Doering et al. 2015). In the present study, the luciferase reporter gene assays were performed essentially as described in (Grun et al. 2002; Lille-Langoy et al. 2015), however, transfection was performed with the TransIT-LT1 reagent (Mirus BIO, USA) as described by the supplier. In brief, we created plasmids encoding fusion proteins of the high-Antarctic Notothenioid Ahr2 LBD region (corresponding to AA238-414 in zebrafish Ahr2, UniProt: #Q9YGV3) and of yeast Gal4-DNA-binding domain (DBD). The plasmid encoding a fusion protein encoding the GAL4-DNA-binding domain and the LBD of the Atlantic cod AHR (AA221-439) was already available to us (Madsen 2016).

The plasmid containing the Ahr2-GAL4 fusion protein was co-transfected into COS7 cells together with a reporter plasmid (MH100)x4tk luc and a CMV-promoter based plasmid constitutively expressing ß-galactosidase as a transfection control (Forman et al. 1995; Blumberg et al. 1998). The COS7 cells were maintained in phenol red Dulbecco's modified Eagle medium (DMEM), supplemented with 10% foetal bovine serum (FBS), 4 mM L-glutamate, 1 mM sodium pyruvate at 37 °C with 5% carbon dioxide (CO_2_). Penicillin and streptomycin at concentrations of 100 U*mL^−1^ were added to prevent microbial contamination in the growth media. Cells were seeded in 96-well plates at densities of 5*10^3^ cells per well and cultivated for 24 h. Cells were then transfected with the luciferase reporter, the ß-galactosidase control plasmids and plasmids encoding high-Antarctic Notothenioid Ahr2 LBD-GAL4-DBD fusion proteins. After 24 h of incubation, cells were exposed to the test compounds dissolved in DMSO at seven different final concentrations in phenol red-free DMEM supplemented with 10% heat-inactivated, charcoal-resin stripped FBS. The concentrations of BaP ranged from 0.0013 to 20 µM, of BNF from 0.0003 to 4 µM and of chrysene from 0.0032 to 50 µM, based on preliminary studies with Atlantic cod elaborating suitable compound concentration ranges and exposure durations (Aranguren-Abadía et al. 2020). Twenty-four hours post-exposure, the COS7 cells were lysed and enzyme activities of luciferase and ß-galactosidase measured as luminescence and absorbance, respectively (EnSpire multimode plate reader, Perkin Elmer, USA). Each compound was measured in triplicate wells and five different assays. The activities of ß-galactosidase resulting from the constitutive expression of the control plasmid were monitored and used to correct for differences in transfection efficiency between the wells.

Prior to the start of the luciferase reporter gene assay with our Ahr-fusion proteins, viability assays were performed to make sure that non-toxic concentrations of the individual test compounds were used. This cytotoxicity assay basically followed the experimental procedure of the luciferase reporter gene assay, without transfection of the reporter-, effector- and control plasmids. In the end, cell viability was measured with 5-carboxyfluorescein diacetate, acetoxymethyl ester (CFDA-AM) to test for membrane integrity, and with resazurin to measure cell metabolism, as fluorescence in a EnSpire multimode plate reader (Perkin Elmer, USA) as previously described (Blanco et al. 2018).

The activation of Ahr in cells exposed to the toxicants was expressed as transfection efficiency corrected luciferase activities (normalized relative luciferase units) relative to activities in solvent control cells (fold-induction), thereby accounting for any (unlikely) Ahr background activities in the assay as such. Dose–response curves were fitted by linear regression analysis in Prism 5.02 (GraphPad Software, Inc., La Jolla, USA). The statistical difference of the cells exposed to compounds compared to control (DMSO) was evaluated by ANOVA followed by a post hoc test (Dunnett) (Software: GraphPad Prism). The statistical difference between the high-Antarctic Notothenioid Ahr LBD and Atlantic cod Ahr LBD was calculated by ANOVA. A *p* ≤ 0.05 was considered to be significant. Data are presented as means ± standard error of the mean (± sem).

### Isolation of hepatocytes

Cells from about 2 g liver tissue were isolated following a protocol modified after (Mommsen et al. 1994; Segner 1998) and a protocol for the isolation of hepatocytes from *G. morhua* after Stapp et al. (2015). All isolation buffers and media were chilled on ice prior to use. The liver was excised and immediately placed in Hanks medium containing 30 mM NaCl and 10 mM 4-(2-hydroxyethyl)-1-piperazineethanesulfonic acid (HEPES). The liver was then flushed with Hanks medium substituted with 30 mM NaCl, 10 mM HEPES and 5 mM Ethylenediamine Tetraacetic Acid (EDTA). Afterwards, it was perfused and digested for 1–1.5 h with Hanks medium containing 30 mM NaCl, 10 mM HEPES, 1% w*v^*−1*^ BSA and 1500 U*ml^−1^ collagenase type 1A. After digestion time, the digestion process was stopped by adding 10% (v*v^*−1*^) fetal calf serum (FCS). Subsequently, hepatocytes were filtered through a series of nylon mesh (250 µm, 105 µm and 50 µM) followed by centrifugation at 100 g, 0 °C, for 3 min. The concentrated hepatocytes were washed three times in Hanks substituted with 2 mM CaCl2 and resuspended in Leibovitz L15 (+ glutamine, Sigma L4386) containing 15 mM NaCl and 10 mM HEPES. Cells were stored on ice on a shaking desk for at least one hour prior to experimentation. Cell numbers were assessed in a Fuchs-Rosenthal haemocytometer dish and cell viability was determined by Trypan blue exclusion.

In preliminary experiments, we observed increased mortality of the cells six hours after isolation. We thus chose the maximum duration of the subsequent assays accordingly.

### Measurement of oxygen consumption

Cell-respiration was measured in vitro in airtight 0.5-ml glass respiration chambers containing magnetic stirrers from Loligo Systems (Viborg, Denmark). Oxygen concentrations within the chambers were detected once per minute with fibre-optic oxygen sensors (Polymer Optical Fiber PSt3 & Oxygen Sensor Spots, PreSens—Precision Sensing GmbH, Germany) connected to an OXY-4 multi-channel Oxygen Meter (PreSens, Germany). Before each measurement, the oxygen probes were calibrated at 100 and 0% oxygen concentration within the respiration chambers containing the respiration medium chilled at 0.5 °C. The respiration chambers were temperature controlled at 0.5 °C by a water bath, three chambers were measured in parallel.

Cell solutions were diluted to achieve final cell counts of 2.5 million cells per chamber. Cells were incubated in the temperature-controlled glass respiration chambers for one hour prior to measurement at the respective measurement conditions to ensure that BaP was metabolized by the cells.

In vitro studies on non-polar fish demonstrated intrinsic clearance of BaP in isolated hepatocytes or liver microsomal fractions already at concentrations of 5 µM BaP and below (Fay et al. 2014; Möller et al. 2014b). To avoid potential cytotoxicity effects by BaP in our experiments, but to make sure BaP metabolism was induced in the isolated hepatocytes, we exposed isolated hepatocytes to 0.5, 1, 5, 10 and 20 µM BaP for a period of 24 h in preliminary experiments with three samples of *T. loennbergii* and *C. hamatus* each. Cytotoxicity and viability were analysed with Trypan blue exclusion and a Neutral red assay (Sigma) (Weyermann et al. 2005). The increasing BaP concentrations revealed no significant differences in cell viability, mortality or any significant cytotoxicity effects (data not shown), and 10 µM BaP was selected as concentration for the acute BaP exposure assays.

Oxygen consumption was recorded in control cells, cells + DMSO (solvent control) and cells + 10 µM BaP. Cycloheximide was used to measure energy allocation to protein biosynthesis. The cycloheximide concentration was applied to inhibit protein biosynthesis at the lowest effective dose (100 µM) according to Stapp et al. (Stapp et al. 2015). Fourty-five minutes after start of measuring the cellular respiration, the inhibitor was added.

The test compounds and inhibitor were dissolved in DMSO; added volumes did not exceed 1% of the final assay volume to avoid cytotoxicity. All chemicals were purchased from Sigma Aldrich, Switzerland.

Oxygen consumption rates were calculated using the slope of oxygen depletion within the chambers. Hepatocyte respiration is displayed as MO_2_ in pmol O_2_ min^−1^ 10^6^ cells^−1^.

The hepatocyte respiration data were analysed using Prism 5.02 (GraphPad software, Inc., La Jolla, CA, USA). All data were tested for normality (Kolmogorov–Smirnov) and homogeneity of variance. Using analysis of variance (ANOVA) followed by Tukey post-tests or pairwise comparison with two-tailed *t*-tests (Students), we evaluated statistical differences between control and BaP-exposed cells and species differences. The following parameters were analysed for statistical differences: hepatocyte respiration (MO_2_ in pmol O_2_ min^−1^ 10^6^ cells^−1^), protein biosynthesis (as % of control respiration (0 °C, no BaP)) and BaP metabolism (as % of control respiration). A *p* ≤ 0.05 was considered as significant differences. All data are presented as means ± standard error of the mean (± sem).

## Results

### Sequencing and cloning of high-Antarctic fish Ahr

**Fig. 1:**
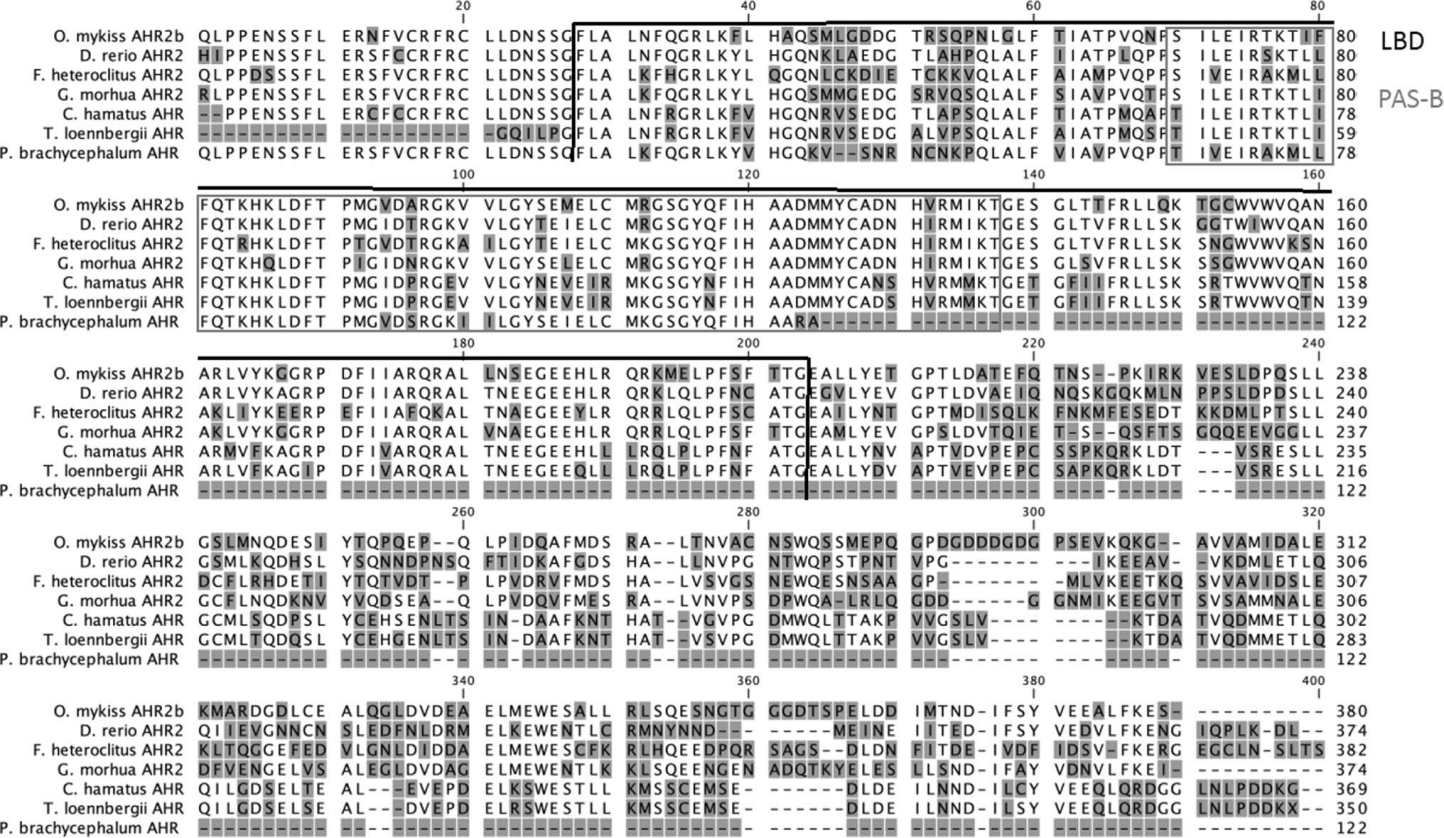
Alignment of the ligand binding domain (LBD) and PAS-B amino acid sequences of rainbow trout, zebrafish, killifish, Atlantic cod and high-Antarctic Notothenioid Ahrs. The amino acid sequences were aligned using CLC Main Workbench. Differences in amino acid sequences are shaded in grey. The LBD is highlighted by black brackets, the PAS-B domain is depicted by a grey box. GenBank accession numbers are: rainbow trout (*Oncorhynchus mykiss*) Ahrβ *#*NP001117724.1, zebrafish (*Danio rerio*) Ahr2 #NP571339.1, killifish (*Fundulus heteroclitus*) Ahr2 #AAC59696.3, *Gadus morhua* Ahr2, *Chionodraco hamatus* Ahr, *Trematomus loennbergii* Ahr, Antarctic eelpout (*Pachycara brachycephalum*) Ahrβ #KY747527.1

Using primers located in conserved regions (bHLH and PAS-B) of teleost fish Ahr, we were able to amplify and clone cDNA fragments of the predicted size (~ 900 bp) of both *C. hamatus* and *T. loennbergii*. The nucleotide sequences of these cloned PCR products were highly similar to sequences of the Ahr of other teleost fish species (BlastN at ncbi.nml.nig.gov; Figure S1 and S2, supplementary). For both high-Antarctic species, only a single Ahr sequence was obtained in several cloning approaches. The obtained cDNA and deduced amino acid sequences covered the LBD and PAS-B domain (Genbank accession numbers: *T. loennbergii*: #MG825103, *C. hamatus*: #MG825104; Fig. 1).

The two amino acid sequences reported here for the two high-Antarctic fish species display 95% sequence identity to each other. An alignment with other fish Ahrs revealed conserved regions and similarities in the LBD amino acid sequences. Amongst others, the Ahr LBD of *C. hamatus* and *T. loennbergii* displays 71% identity to *G. morhua* and 65% identity to killifish Ahr2. *C. hamatus* showed 60% identity and *T. loennbergii* 58% identity to zebrafish Ahr2 (Fig. 1).

**Fig. 2:**
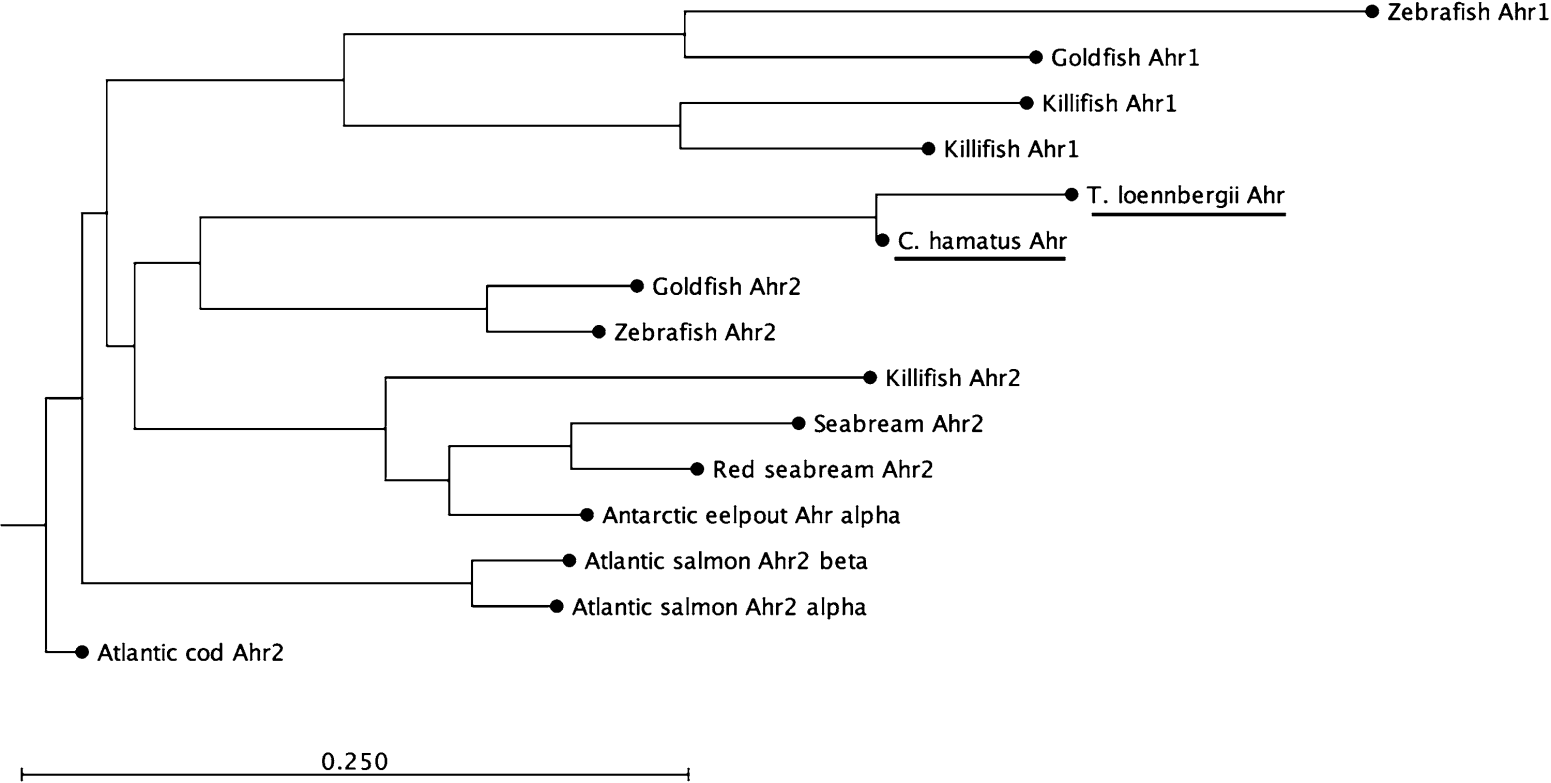
Phylogenetic analysis of fish Ahr amino acid sequences. The amino acid sequences were aligned using CLC Main Workbench. The phylogenetic tree was constructed by the neighbour-joining method using CLC Main Workbench, with the branch lengths corresponding to the evolutionary distance between sequence clusters. The GenBank accession of the sequences are as follows: seabream Ahr2 #AAN05089, killifish (*Fundulus heteroclitus*) Ahr1 #O57452, killifish (*Fundulus heteroclitus*) Ahr2 #AAC59696, goldfish (*Carassius auratus*) Ahr1 #ACT79400, goldfish Ahr2 #ACT79401, zebrafish (*Danio rerio*) Ahr1 #NP571103, zebrafish (*Danio rerio*) Ahr2 #NP571339, red seabream (*Pagrus major*) Ahr2 *#*BAE02825, Atlantic salmon (*Salmo salar*) Ahr1 #NP_001117158.1 Atlantic salmon (*Salmo salar*) Ahr2α #NP_001117156, Antarctic eelpout (*Pachycara brachycephalum*) Ahr2α #KY747528, Atlantic cod (*Gadus morhua*), Chionodraco (*Chionodraco hamatus*) Ahr2 and Trematomus (*Trematomus loennbergii*) Ahr2

To elucidate the relationship of the high-Antarctic Notothenioid Ahr LBDs to other fish Ahr proteins, we performed a phylogenetic analysis of selected teleost fish Ahrs and our partial Ahr amino acid sequences (Fig. 2). The analyses revealed that the Ahr of the high-Antarctic fish species grouped together with Ahr2 amino acid sequences of other fish. These grouping results are strongly supported by bootstrap analysis (100%). Based on this phylogenetic analysis and the bootstrap support, we designate the Ahr LBDs of *C. hamatus* and *T. loennbergii* to belong to the Ahr2 clade.

### Concentration-dependent activation of high-Antarctic fish Aryl hydrocarbon receptor ligand binding domains

By using the GAL4-based reporter gene assay, we measured the agonistic transactivation of the Ahr2 LBDs from *C. hamatus* and *T. loennbergii* as a significant change in luciferase activity in cells treated with compounds relative to solvent (DMSO)-treated cells*.* For comparison, we also assessed the activation of the Ahr2 LBD from *G. morhua*. A cytotoxicity test ensured that the cells were not exposed to cytotoxic (i.e. lethal) concentrations of the test compounds (Table S2, supplementary).

**Fig. 3:**
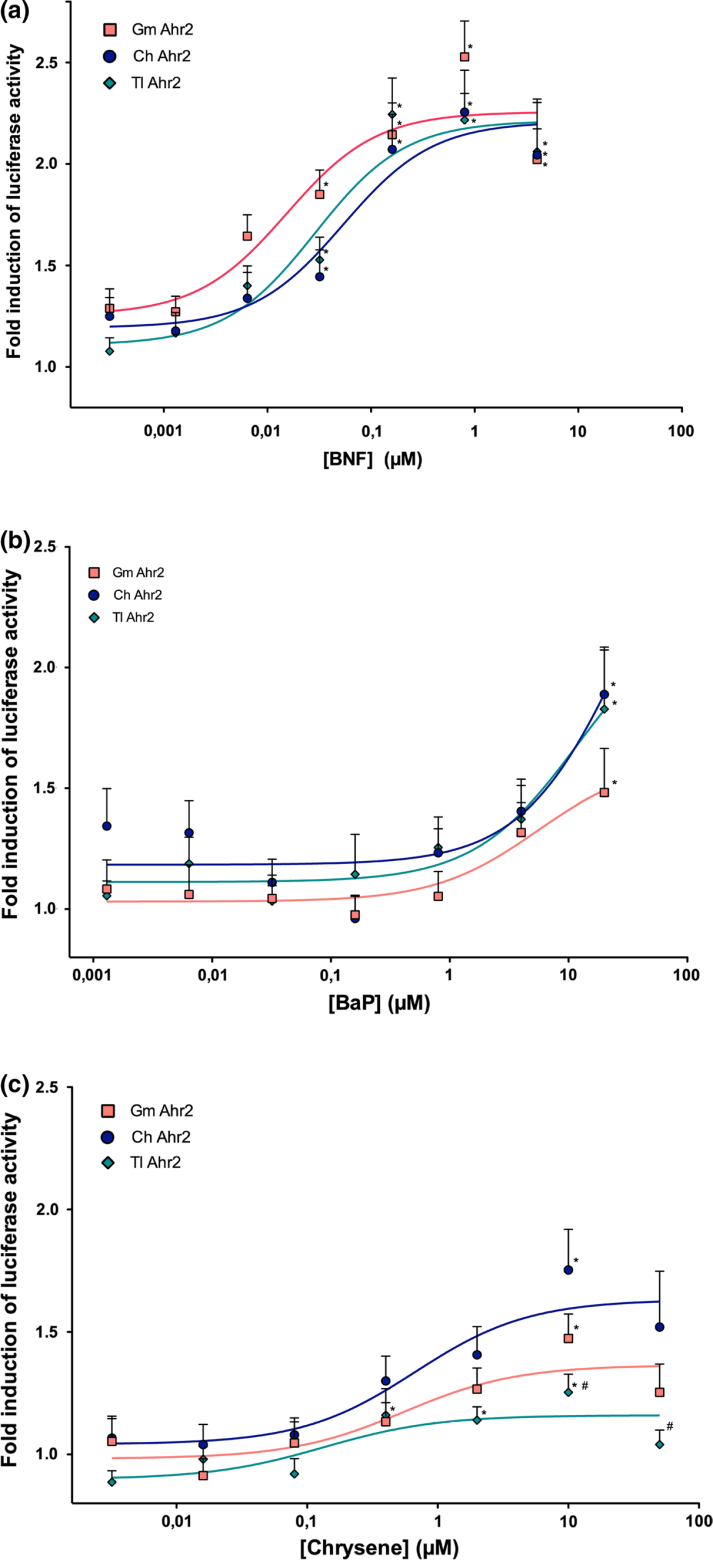
Ligand activation dose–response curves of Atlantic cod (*Gadus morhua*) (*n* = 3) Ahr2 ligand binding domain (Gm Ahr2), *Chionodraco hamatus* (*n* = 3) Ahr ligand binding domain (Ch Ahr LBD) and *Trematomus Loennbergii* (*n* = 3) Ahr ligand binding domain (Tl Ahr LBD). The ligand activation of Ahr by selected test compounds (**a = beta-naphthoflavone (BNF), b = Benzo[a]pyrene (BaP), c = chrysene**) is reported as fold-increase in luciferase activity in cells exposed to the test compound over cells exposed to solvent (dimethyl sulfoxide, not shown). Each data point represents the mean of triplicate wells measured in five independent experiments (± sem). The dose–response curves were fitted by non-linear regression (GraphPad Prism). * displays a statistically significant difference in luciferase activities in compound-exposed compared to dimethyl sulfoxide -treated cells (ANOVA, *p* ≤ 0.05. Detailed statistics are given in Table S3, supplementary). # shows a significant difference in luciferase activities compared to the luciferase activity of *Chionodraco hamatus* (ANOVA, *p* ≤ 0.05. Detailed statistics are given in Table S3, supplementary)

The results of the in vitro ligand activation of the Ahr LBD are summarized in Table S3 (supplementary) and Fig. 3, where the relative increase in luciferase activity is displayed for the five highest concentrations of each compound. All three compounds tested in the GAL4-LBD based luciferase reporter gene assay caused a concentration-dependent induction in luciferase activity in the high-Antarctic fish and in Atlantic cod. Beta-naphthoflavone caused the strongest induction of luciferase activity, which was significantly higher than in the DMSO-treated cells at all concentrations above 0.032 µM (Table S3, supplementary and Fig. 3). BaP significantly increased luciferase activity compared to the DMSO-exposed cells only at the concentration of 20 µM in all three species, and higher concentrations were not tested.

The luciferase activity rose with increasing chrysene concentration and displayed the highest, significantly increased luciferase activity at 10 µM in all three species (for detailed statistics, see Table S3, supplementary). At 50 µM, the luciferase activity decreased already, indicating a slight inhibitory effect at such high concentrations, although no significant cytotoxicity was observed (Table S2, supplementary). A species comparison between the three fish revealed that the luciferase activity produced by transactivation of the Ahr LBD of *C. hamatus* was significantly higher than in *T. loennbergii* at 10 and 50 µM chrysene, but not different to *G. morhua* with this assay (for detailed statistics, see Table S3, supplementary)*.*

### Effect of benzo[a]pyrene on the hepatocyte respiration and costs for benzo[a]pyrene metabolism

In preliminary experiments, we could show that DMSO (the inhibitors’ solvent), had no significant effect on the hepatocyte respiration rates, compared to the control hepatocytes.

**Fig. 4:**
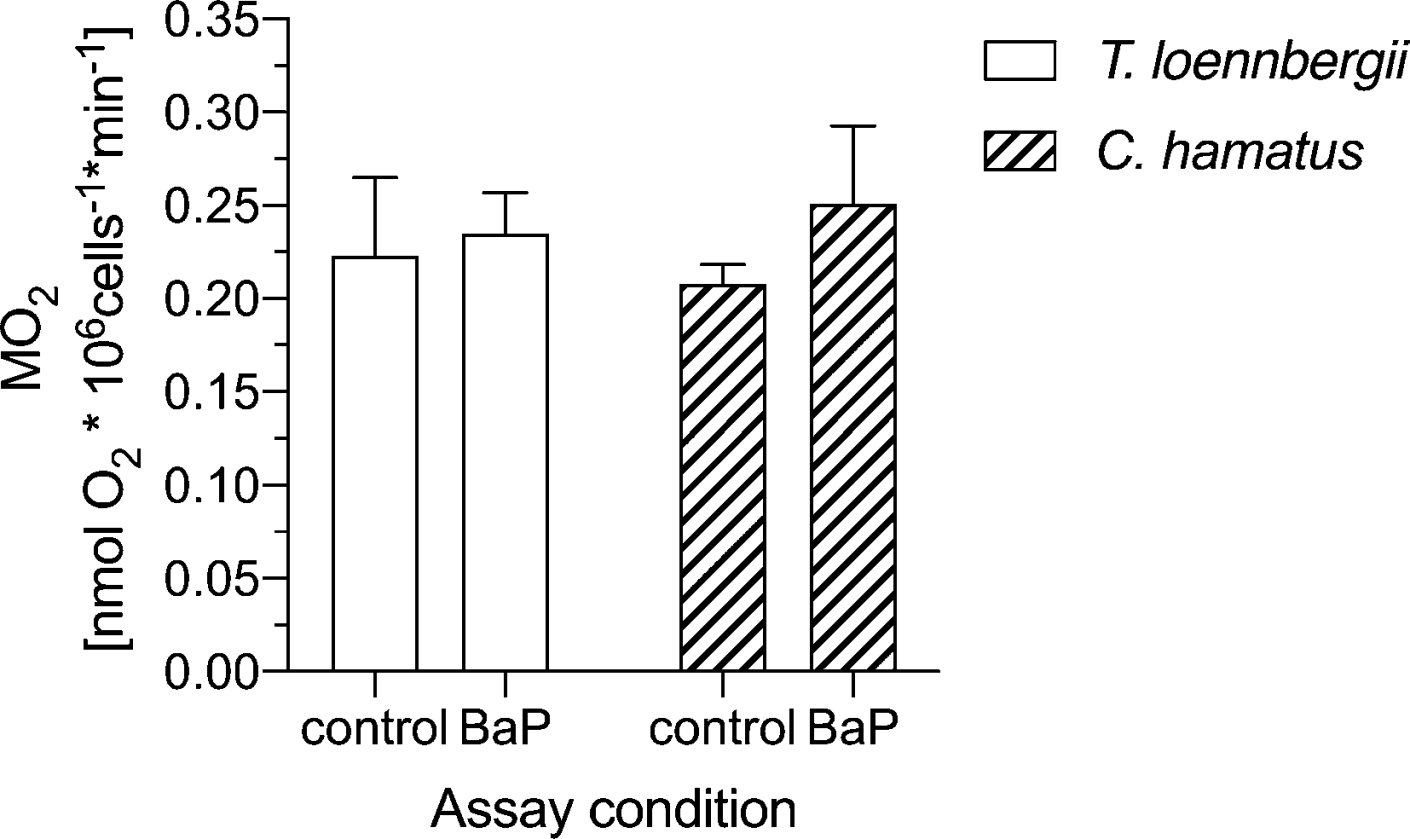
Oxygen consumption (MO_2_) of liver cells of *Trematomus loennbergii* (*n* = 8, plain bars) and *Chionodraco hamatus* (*n* = 6, striped bars). Hepatocyte MO_2_ was measured under control and BaP-exposed conditions at 0 °C. Values are displayed as means ± sem

Figure 4 displays the hepatocyte respiration rate in *T. loennbergii* and *C. hamatus* at 0 °C in control and BaP-exposed cells. We neither measured a significant difference in hepatocyte respiration rate in control *vs.* BaP-exposed cells, nor between the red- and white-blooded species. In *T. loennbergii,* we measured a hepatocyte respiration rate of 0.22 ± 0.04 nmol O_2_ *10^6^ cells^−1^ min^−1^ (*n* = 8) in the control cells, and 0.23 ± 0.02 nmol O_2_ *10^6^ cells^−1^ min^−1^(*n* = 8) in the BaP-assay. In the control and BaP-exposed cells of *C. hamatus*, we measured a hepatocyte respiration of 0.21 ± 0.01 and 0.25 ± 0.04 nmol O_2_ *10^6^ cells^−1^ min^−1^ (*n* = 6), respectively.

### Protein metabolism in benzo[a]pyrene exposed hepatocytes

**Fig. 5:**
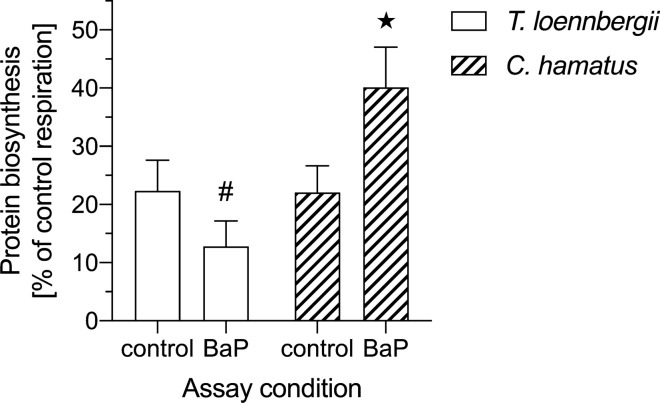
Energy expenditure for protein biosynthesis in control (sham) and BaP-exposed liver cells of *Trematomus loennbergii* (*n* = 8, plain bars) and *Chionodraco hamatus* (*n* = 6, striped bars) assayed at 0 °C. Protein biosynthesis is displayed as % of control (sham) respiration. The # depicts a significant difference (unpaired *t*-test, *t*_6_ = 3.536, *p* = 0.012) to *Chionodraco hamatus* at the respective assay conditions. The asterisk displays a significant difference (unpaired *t*-test, *t*_4_ = 2.163, *p* = 0.048)) to the protein biosynthesis in BaP-exposed liver cells of *Trematomus loennbergii*. Values are means ± sem

Figure 5 depicts the metabolic costs dedicated to protein biosynthesis as % of control respiration in isolated hepatocytes of *T. loennbergii* and *C. hamatus*, which was 22% in the control hepatocytes of both species.

In *T. loennbergii*, the fractional oxygen demand for protein biosynthesis accounted for 22 ± 5% (*n* = 8) in the control hepatocytes and for 13 ± 4% (*n* = 8) in the BaP-exposed cells.

In the control assay of *C. hamatus*, 22 ± 4% (*n* = 6) of the total hepatocyte respiration was dedicated to protein synthesis*.* This fraction rose significantly (Unpaired *t*-test, *t*_4_ = 2.163, *p* = 0.048) to 40 ± 7% (*n* = 6) in the BaP-exposed hepatocytes.

## Discussion

### Aryl hydrocarbon receptor sequencing and expression in high-Antarctic fish species

In this paper, we identified and cloned a partial gene sequence of the Ahr corresponding to the Ahr LBD in the high-Antarctic Notothenioids *C. hamatus* and *T. loennbergii* and demonstrated that the Notothenioid Ahr LBDs are activated by PAHs in the GAL4-based reporter gene assay. Furthermore, we demonstrated that PAHs leave a metabolic mark in hepatocytes from the two Antarctic fish species.

Fish, in contrast to mammals, do not have a single Ahr but show a high diversity of *ahr* genes. Teleost fish possess two Ahr clades (Ahr1 and Ahr2), which can comprise several isoforms (Hahn et al. 2006). A sequence comparison of the *C. hamatus* and *T. loennbergii* Ahr LBD amino acid sequence with other piscine Ahrs revealed high identities with e.g. *F. heteroclitus* Ahr2b and other fish Ahr-like sequences. This similarity was further supported by a phylogenetic analysis, which showed that the Ahrs of *C. hamatus* and *T. loennbergii* cluster together with the Ahr2 clade of teleost fish. Furthermore, the Ahr LBD regions that we cloned from liver cDNA contained highly conserved regions when compared to other fish Ahr LBDs (Fig. 1), demonstrating that high-Antarctic Notothenioids express *ahr2* in the liver. Indeed, previous studies report that *ahr2* is expressed at particularly high levels in liver tissues of fish, and in some species also at much higher levels than *ahr1* (Abnet et al. 1999; Hansson and Hahn 2008; Aranguren-Abadía et al. 2020). This may explain why we could not obtain other *ahr* isoforms in *C. hamatus* or *T. loennbergii*, despite several cloning attempts. Nonetheless, we cannot exclude that *ahr1* is expressed in high-Antarctic Notothenioids as well. To our knowledge, to date only one study measured the expression of *ahr* in an Antarctic fish species, the Antarctic eelpout (Strobel et al. 2018), and no functional information of the Ahr exists for any Antarctic teleost species. Importantly, the expression of the Ahr LBD in species like Antarctic eelpout or its presence in the investigated high-Antarctic fish does not reflect its capacity for ligand activation by Ahr agonists.

### Activation of high-Antarctic fish Aryl hydrocarbon receptor by polycyclic aromatic hydrocarbons

Our results of the luciferase reporter gene assays revealed that the Ahr LBD of both high-Antarctic species and Atlantic cod responded to the exposure to BaP, BNF and chrysene by an increase in luciferase activity and in a dose-dependent manner. Despite the differences in the protein primary sequence and potential conformation differences of the Ahr LBD of high-Antarctic fish (95% sequence identity between the two species) and Atlantic cod (52 substitutions out of 177 amino acids), we did not observe significant differences in the activation characteristics of the Ahr LBD between the high-Antarctic fish and Atlantic cod in the assays using BaP and BNF as ligands. Thereby, the results of the luciferase reporter gene assay suggest that Ahr is an active, ligand-activated transcription factor in high-Antarctic Notothenioids. Even though the differential ligand binding affinities appear to depend on the ligand binding cavity in fish of the piscine Ahr (Fraccalvieri et al. 2013), the GAL4-based luciferase assay with the Atlantic cod Ahr LBD is less sensitive in comparison to a full-length Ahr and Arnt/XRE-based luciferase assay. Notably, the efficacy and sensitivity of Ahr towards BaP were shown to be considerably higher in the full-length assay, which is also in accordance with prior research on fishes (Aranguren-Abadía et al. 2020). Thus, using the full-length Ahr sequences in a similar assay could potentially reveal masked differences in sensitivities amongst the high-Antarctic fish and Atlantic species. Moreover, the determinants responsible for causing differences in Ahr sensitivities amongst fish species are currently not elucidated (Doering et al. 2013). In contrast, it is well documented in birds that even small deviations in the amino acid sequence of the LBD drive species-specific differences in Ahr sensitivities (Karchner et al. 2006). Interestingly, studies on the mammalian Ahr indicate that the C-terminal transactivation domain may play a larger role than the LBD in determining ligand sensitivity (Boutros et al. 2008; Wang et al. 2013). Thus, comparative and functional analyses conducted solely on the Ahr LBDs from fish must be interpreted with caution as differences in sensitivities and activation profiles amongst fish species may originate from features present elsewhere in the Ahr protein structures. Due to these limitations of the GAL4-LBD assay, it is important to emphasize that it does not necessarily reflect quantitative differences in ligand activation and sensitivities between high-Antarctic fish and Atlantic cod (Wang et al. 2013).

The results of the GAL4-LBD based reporter gene assay are also in line with previous findings in Antarctic Notothenioids from the gene expression or enzymatic level, which showed that BaP exposure causes an increase in *ahr2* and *cyp1a* expression and the enzymatic activity of Cyp1a, both underlining a responding Ahr pathway to PAHs (Regoli et al. 2005; Strobel et al. 2015). Yet, we observed differences between *C. hamatus* and *T. loennbergii* in the Ahr activation at the two highest chrysene concentrations tested in the reporter gene assays, in contrast to the responses we obtained for the ligands BaP and BNF. As such, a different in vitro induction of the Ahr and its downstream processes in response to certain PAHs is a first indicator of putative differences in the biotransformation metabolism between red- and white-blooded Notothenioids, and potentially the associated metabolis costs.

Despite these observed differences in the ligand activation by high doses of chrysene in *C. hamatus* and *T. loennbergii*, the overall response of the Ahr LBD to organic compounds was hardly distinct between the high-Antarctic fish and Atlantic cod in the reporter gene assay.

However, although we could only use the Ahr LBD in the assays, the measurement of Ahr related activation represents a great potential to provide a first estimate of the sensitivity of species to dioxin-like compounds. Especially considering the difficulties in obtaining various Antarctic fish species in a living state, this type of experiment offers an excellent opportunity for future ecotoxicology studies and to assess species differences in sensitivity to environmental contaminants in the Antarctic. The demand for such type of investigations will increase under the conditions of climate change, placing Antarctic fish under even higher numbers of stressors, resulting in a complex multiple stressor scenario. Here, the broad spectrum of Ahr functions has to be considered, as it is likely acting as a convergence point where various endogenous and external signals are integrated, including nutritional factors or molecules of the microbiome (Segner et al. 2021). Obtaining the full-length Ahr of various Antarctic fish species would be crucial here to develop a robust relationship between Ahr activation and stressor sensitivity.

Importantly, Antarctic fish possess generally low metabolic rates and only a slow xenobiotics metabolism, in comparison to temperate zone fish (Clarke 1991; Focardi et al. 1995; Möller et al. 2014a; Strobel et al. 2015). In the end, a functioning but slower xenobiotics metabolism and excretion would have consequences for e.g. the exposure time, bioconcentration and bioaccumulation of organic pollutants in the lipid-rich tissues of the Antarctic fish, and therefore the toxicity of individual POPs. As pointed out by Strobel et al. (2015), despite a functional Ahr pathway, the low biotransformation rates that appear to exist in Antarctic fishes implicate a high bioaccumulation of lipophilic contaminants, thus potentially putting Antarctic fish at risk even at low environmental exposure concentrations.

### Effects of of benzo[a]pyrene on hepatocyte energy metabolism

Energy investment and allocation is a common functional response to environmental stressors at all levels of organization, and several studies underline shifts in an animal’s energy budget as long-term consequence to environmental stress (Pörtner 2012; Sokolova et al. 2012). Many Notothenioids, however, already reside at the upper end of their thermal tolerance range, implying that they are energetically limited and their physiological performance is highly susceptible to additional environmental stress (Pörtner 2012; Pörtner and Lannig 2009). Thus, energetic limitations are a critical factor for their physiological capabilities to cope with environmental stressors such as anthropogenic pollutants, especially if they are altering the energy investment for functions such as growth or reproduction (Sandersfeld et al. 2016). In terms of energy production and storage, it is the liver which plays a central role and serves as hub of several metabolic pathways (Strobel et al. 2013). Moreover, it is a central organ in the Ahr-mediated metabolism of xenobiotics (Hinton et al. 2008). Indeed, a few studies already report an additional fraction of energy that is needed for detoxification processes (Ng and Gray 2011; Manciocco et al. 2014), and a few in vivo studies demonstrated altered hepatocyte metabolism in response to xenobiotics exposure in non-polar fish (Bains and Kennedy 2005; Nault et al. 2012). Thus, there is a need to determine the metabolic capacities to cope with anthropogenic pollutants. Assessing hepatic metabolism in Antarctic fish is therefore of high value to evaluate their capacities to deal with emerging contaminants, and if resource competitions could arise with other energy-demanding processes (French et al. 2009).

The hepatocyte respiration rates of *C. hamatus* and *T. loennbergii* that we measured under control conditions in this study were within the same order of magnitude as hepatocyte respiration rates of other *Trematomus* and *Lepidonotothen* species, the Antarctic eelpout *P. brachycephalum* (Langenbuch 2003; Mark et al. 2005), and to respiration rates of *G. morhua* (Stapp et al. 2015), rainbow trout or goldfish (Krumschnabel et al. 1997; Wieser and Krumschnabel 2001). In fact, various studies on Antarctic Notothenioids suggest that there is no difference in their tissue oxidative capacities compared to cold-eurytherm fish, reflecting the perfect cold-adaptation in polar fishes (Johnston et al. 1998; Pörtner et al. 2000). Furthermore, the hepatocyte respiration was at the same level in *T. loennbergii* and *C. hamatus*, in line with previous reports on similar whole animal respiration rates in haemoglobin-less and red-blooded Antarctic fish (Ralph and Everson 1968).

Next, we observed no significant effect of BaP on the respiration rate of hepatocytes from *T. loennbergii* or *C. hamatus*. The existing data on the impact of Ahr-binding toxicants on cellular metabolism is indeed conflicting: whilst studies with rainbow trout hepatocytes reported an increase in oxygen consumption rate after exposure to pyrene (Bains and Kennedy 2005), other studies found that isolated primary hepatocyte cultures of rainbow trout did not change respiratory activity in response to PCB 77 and 126 (Nault et al. 2012). Theoretically, one would expect opposite findings, as PCB 77 and 126 are slowly metabolized, whereas BaP is rapidly metabolized so that the cost of biotransformation should be higher in the BaP-exposed trout hepatocytes. Probably, the energetic costs of biotransformation in the overall metabolic costs of hepatocytes are not high enough to cause significant changes in whole-cell respiration. This should be even more the case with Antarctic Notothenioid fish, which display particularly low rates of biotransformation (Strobel et al. 2015).

### Effects of benzo[a]pyrene on hepatic protein biosynthesis

An induction of biotransformation by xenobiotics implicates the need for increased cellular protein synthesis in order to establish elevated levels of the relevant metabolic enzymes. Thus, we were interested to learn if the costs associated with BaP exposure are different if we consider specifically protein synthesis rather than the overall cellular metabolism.

Concerning the share of respiration dedicated to protein synthesis, hepatocytes of *T. loennbergii* and *C. hamatus* showed very similar values to those reported previously for Antarctic and non-polar fish species, including *G. morhua* (Krumschnabel et al. 1997; Langenbuch and Pörtner 2004; Mark et al. 2005; Lewis et al. 2015; Stapp et al. 2015). In BaP-exposed cells, the metabolic costs for protein synthesis increased significantly in *C. hamatus* to up to 40%. In contrast, the respiration dedicated to protein synthesis in *T. loennbergii* tended to decrease to 13%. Both values are either higher or lower than the costs for protein synthesis reported previously for Antarctic fish, in which they ranged from 18 to 37% (Langenbuch and Pörtner 2004; Mark et al. 2005; Lewis et al. 2015). Even though not obvious when looking at overall hepatocyte respiration in BaP-exposed cells of *C. hamatus*, BaP thus had a clear effect on the allocation of cellular energy, indicating shifts towards protein synthesis. Such elevated protein synthesis, i.e. an increased translation efficiency could indeed support the detoxification of xenobiotics, including the Ahr pathway and downstream enzymatic biotransformation processes (Hinton et al. 2008). However, such metabolic shifts could also reduce the amount of energy available for other processes of the aerobic metabolism such as lipid or glycogen biosynthesis (Sidell et al. 1987; Windisch et al. 2011). Finally, this would lead to a mobilization of the liver energy stores such as glycogen or lipid, to compensate for the reduced amount of energy available for these pathways (Strobel et al. 2013).

In contrast to *C. hamatus, T. loennbergii* showed a different pattern with a tendency to a slightly reduced amount of protein metabolism, potentially to foster other processes. Indeed, protein synthesis is proposed to be the most sensitive process to changes in cellular energy supply (Wieser and Krumschnabel 2001). Accordingly, an increased demand by other energy-demanding processes could negatively affect the costs dedicated to protein synthesis in BaP-exposed hepatocytes of *T. loennbergii.* To this end, the shifts in metabolic pathways that we observed for *C. hamatus* and tendentially for *T. loennbergii* would affect energy stores of both the white- and red-blooded Notothenioids, and consequently the whole animal performance in the long run.

## Conclusion

With the results of the present study, we provide initial evidence that BaP can transactivate Ahr2s from Antarctic Notothenioids, suggesting that PAHs activate the Ahr signalling pathway in these fish. This finding is of relevance as it indicates that functional losses of elements of the defensome such as the heat shock response and Pxr, as observed in a number of fish species from extreme environments (Eide et al. 2018), do not extend to all members of the cellular defensome.

Further evidence for the functional activation of the Ahr pathway in Antarctic Notothenioids comes from the observation that it is associated with increased energy allocation to hepatocellular protein synthesis. Remarkably, the latter response display differences between the red- and white-blooded Notothenioids. The question remains what consequences the BaP-induced shift in the cellular energy allocation might have for the overall energy metabolism of Notothenioids and thus the overall performance of the animals in the long term.

## Supplementary Information

Below is the link to the electronic supplementary material.

Supplementary file1 (DOCX 26 kb)
