# Supplementary material for: Xenobiotic metabolism and its physiological consequences in high-Antarctic Notothenioid fishes
Source: Polar Biol. 2021 Dec 26;45(2):345–58. doi: 10.1007/s00300-021-02992-4 (PMC8818001; doi:10.1007/s00300-021-02992-4)
Supplement: Supplementary file 1 — Supplementary file1 (DOCX 26 kb) [file 300_2021_2992_MOESM1_ESM.docx]

**Supplementary**

**Xenobiotic metabolism and its physiological consequences in high-Antarctic Notothenioid fishes**

**Polar Biology**

Anneli Strobel^*,a^, Roger Lille-Langøy^b,e^, Helmut Segner^c^, Patricia Burkhardt-Holm^d^, Anders Goksøyr^b^ and Odd André Karlsen^b^

*^a^ Alfred Wegener Institute Helmholtz Centre for Polar and Marine Research, Am Handelshafen 12, 27570 Bremerhaven*

*^b^ Department of Biological Sciences, University of Bergen, Thormøhlensgate 53B, N-5006, Bergen, Norway*

*^c^ Centre for Fish and Wildlife Health, Department of Infectious Diseases and Pathobiology, University of Bern, Länggassstrasse 122, CH-3012 Berne, Switzerland*

*^d^ Man-Society-Environment, University of Basel, Vesalgasse 1, CH-4051 Basel, Switzerland*

^e^ *Institute of Marine Research, P.O. Box 1870, Nordnes, NO-5817, Bergen, Norway*

***Corresponding author**:

E-mail: anneli.strobel@awi.de

# Table S1: Oligonucleotide primers for high-Antarctic Notothenioid Aryl hydrocarbon receptor cDNA cloning.

| Primer | Sequence (5’-3’) | Accession nr. */ source |
| --- | --- | --- |
| **PCR** |  |  |
| Ahr fwd | CCGTCCAAGCGGCAT | #NW_011336470.1,  #XM_010778753;  (Karchner et al. 2005) |
| Ahr rev | GCAGCGTGGATGAAGTTG | #NW_011336470.1  #XM_010778753;  (Karchner et al. 2005) |
| **RACE-PCR** |  |  |
| 5R-Ahr-1 | TGGTGACGGCTGAAGGCTAC | #MG825103  #MG825104 |
| 5R-Ahr-2 | CTGGCCGCTGTTTGGCTTTAG | #MG825103  #MG825104 |
| 5R-Ahr-3 | CTGCTCTCCAGGGCTCAGTTC | #MG825103  #MG825104 |
| 3R-Ahr-1 | GAACTGAGCCCTGGAGAGCAG | #MG825103  #MG825104 |
| 3R-Ahr-2 | CTAAAGCCAAACAGCGGCCAG | #MG825103  #MG825104 |

* Accession number of the genes which serves as the basis for the primer design

GSP: Gene specific primer. All primers were synthetized by Microsynth AG (Balgach, Switzerland). Fwd = forward, rev = reverse.

# Table S2: Cell viability in per cent (%), displayed relative to cells exposed to dimethyl sulfoxide (DMSO). Cell viability was assessed with 5-carboxyfluorescein diacetate, acetoxymethyl ester (CFDA-AM) to test for membrane integrity, and with resazurin to measure cell metabolism, as fluorescence in an EnSpire multimode plate reader (Perkin Elmer, USA).

| **Compound concentration (µM)** | Beta-naphtoflavone | Benzo[a]pyrene | Chrysene |
| --- | --- | --- | --- |
| **Resazurin** |  |  |  |
| 50 | na | na | 97.6 |
| 20 | 92.0 | 96.8 | 97.6 |
| 4 | 97.3 | 94.0 | 93.8 |
| 0.8 | 97.9 | 93.3 | 97.6 |
| 0.16 | 94.6 | 38.1 | 98.3 |
| 0.32 | 96.1 | 100 | 98.7 |
| 0.0064 | 93.6 | 97.2 | na |
|  |  |  |  |
| **CFDA-AM** |  |  |  |
| 50 | na | na | 94.1 |
| 20 | 94.5 | 98.5 | 97.1 |
| 4 | 92.2 | 98.2 | 94.7 |
| 0.8 | 99.6 | 96.9 | 93.8 |
| 0.16 | 98.1 | 98.9 | 99.9 |
| 0.32 | 98.7 | 99.5 | 95.6 |
| 0.0064 | 99.6 | 94.4 | na |

**Table S3**: Summary of *in vitro* activation of luciferase activity of *Chionodrao hamatus* (*n* = 3)*, Trematomus loennbergii* (*n* = 3) and Atlantic cod, *Gadus morhua* (*n* = 3) Aryl hydrocarbon receptor 2 ligand binding domain by increasing concentrations of beta-naphtoflavone, benzo[a]pyrene and chrysene, respectively. The results of the luciferase reporter gene assay are expressed as relative luciferase activity in cells exposed to the test compounds compared to the activity in dimethyl sulfoxide (DMSO) exposed (control) cells.

| Compound | *C. hamatus* | *T. loennbergii* | *G. morhua* |
| --- | --- | --- | --- |
| Compound concentration (µM) | Relative to DMSO control | Relative to DMSO control | Relative to DMSO control |
| **BNF** |  |  |  |
| 0.0064 | 1.3 ± 0.1 | 1.4 ± 0.1 | 1.6 ± 0.1 |
| 0.032 | 1.4 ± 0.1 | 1.5 ± 0.1^*^(ANOVA, *F*_7_ = 3.30, *p* ≤ 0.0001) | 1.9 ± 0.1^*^(ANOVA, *F*_7_ = 4.84, *p* ≤ 0.0001) |
| 0.16 | 2.0 ± 0.1^*^(ANOVA, *F*_7_ = 4.92, *p* ≤ 0.0001) | 2.2 ± 0.1^*^(ANOVA, *F*_7_ = 6.93, *p* ≤ 0.0001) | 2.1 ± 0.1^*^(ANOVA, *F*_7_ = 6.53, *p* ≤ 0.0001) |
| 0.8 | 2.3 ± 0.2^*^(ANOVA, *F*_7_ = 5.75, *p* ≤ 0.0001) | 2.2 ± 0.1^*^ (ANOVA, *F*_7_ = 6.79, *p* ≤ 0.0001) | 2.5 ± 1.2^*^(ANOVA, *F*_7_ = 8.73, *p* ≤ 0.0001) |
| 4 | 2.0 ± 0.2^*^(ANOVA, *F*_7_ = 4.79, *p* ≤ 0.0001) | 2.1 ± 0.2^*^(ANOVA, *F*_7_ = 5.99, *p* ≤ 0.0001) | 2.0 ± 0.1^*^(ANOVA, *F*_7_ = 5.83, *p* ≤ 0.0001) |
| **BaP** |  |  |  |
| 0.032 | 1.1 ± 0.1 | 1.0 ± 0.0 | 1.0 ± 0.1 |
| 0.16 | 1.0 ± 0.1 | 1.1 ± 0.2 | 1.0 ± 0.1 |
| 0.8 | 1.2 ± 0.1 | 1.3 ± 0.1 | 1.1 ± 0.1 |
| 4 | 1.4 ± 0.1 | 1.4 ± 0.2 | 1.3 ± 0.1 |
| 20 | 1.9 ± 0.2^*^(ANOVA, *F*_7_ = 5.15, *p* ≤ 0.0001) | 1.8 ± 0.2^*^(ANOVA, *F*_7_ = 4.33, *p* ≤ 0.0001) | 1.5 ± 0.2^*^(ANOVA, *F*_7_ = 3.14, *p* ≤ 0.0001) |
| **Chrysene** |  |  |  |
| 0.08 | 1.1 ± 0.1 | 1.0 ± 0.1 | 1.0 ± 0.1 |
| 0.4 | 1.3 ± 0.1 | 1.1 ± 0.1^*^(ANOVA, *F*_7_ = 2.91, *p* ≤ 0.0001) | 1.1 ± 0.1 |
| 2 | 1.4 ± 0.1 | 1.1 ± 0.0^*^(ANOVA, *F*_7_ = 2.71, *p* ≤ 0.0001) | 1.3 ± 0.1 |
| 10 | 1.8 ± 0.2^*^(ANOVA, *F*_7_ = 3.56, *p* ≤ 0.0001) | 1.3 ± 0.1^*^(ANOVA, *F*_7_ = 3.89, *p* ≤ 0.0001), ^#^ (ANOVA, *F*_3_ = 3.212, *p* ≤ 0.0001) | 1.5 ± 0.1^*^(ANOVA, *F*_7_ = 3.48, *p* ≤ 0.0011) |
| 50 | 1.5 ± 0.2 | 1.0 ± 0.0^#^ (ANOVA, *F*_3_ = 3.084, *p* ≤ 0.0001) | 1.3 ± 0.1 |

Data are presented as the mean of triplicate wells measured in five independent experiments (± sem). The * displays a statistically significant difference in luciferase activities in compound-exposed compared to DMSO-treated cells (ANOVA, *p* ≤ 0.05). The # shows a significant difference in luciferase activities compared to the luciferase activity of *C. hamatus* (ANOVA, *p* ≤ 0.05).
